# Incretin-Based Therapies in Sports: Pharmacological Mechanisms, Performance-Enhancing Potential, and Anti-Doping Implications—A Narrative Review

**DOI:** 10.3390/ijms27146116

**Published:** 2026-07-08

**Authors:** Sandro La Vignera, Rosita A. Condorelli

**Affiliations:** Department of Clinical and Experimental Medicine, University of Catania, 95123 Catania, Italy; rosita.condorelli@unict.it

**Keywords:** incretin therapy, GLP-1 receptor agonists, doping, WADA, athletic performance, semaglutide, liraglutide, body composition, sports pharmacology, anti-doping

## Abstract

Incretin-based therapies, including glucagon-like peptide-1 receptor agonists (GLP-1 RAs) and dipeptidyl peptidase-4 (DPP-4) inhibitors, have revolutionized the management of type 2 diabetes and obesity. Recent evidence suggests these agents may influence athletic performance through effects on body composition, energy metabolism, and cardiovascular function. This narrative review critically evaluates whether incretin therapies could constitute doping under World Anti-Doping Agency (WADA) criteria. We narratively reviewed the literature on incretin pharmacology, metabolic effects relevant to athletic performance, and anti-doping regulations, searching PubMed, Scopus, and Web of Science using terms including “GLP-1 receptor agonists”, “DPP-4 inhibitors”, “doping”, “athletic performance”, “WADA”, and “sports pharmacology”, without date restrictions. GLP-1 RAs (semaglutide, liraglutide, tirzepatide, exenatide) induce substantial weight loss (predominantly fat mass) but also reduce lean mass by 20–30% of total weight loss. Preclinical studies demonstrate enhanced exercise endurance, mitochondrial biogenesis, and glucose uptake via GLP-1R/AMPK signaling. However, clinical trials show no consistent improvement in physical performance in humans. Currently, incretin therapies are not listed on the WADA Prohibited List. While incretin therapies offer theoretical performance-enhancing potential through weight management and metabolic optimization, current evidence does not support classification as doping agents. Continued surveillance is warranted as misuse patterns emerge.

## 1. Introduction

The landscape of sports pharmacology has evolved dramatically over the past decade, with increasing scrutiny of therapeutic agents that may confer competitive advantages beyond their intended clinical indications [1]. Incretin-based therapies, particularly glucagon-like peptide-1 receptor agonists (GLP-1 RAs) and dipeptidyl peptidase-4 (DPP-4) inhibitors, have emerged as transformative treatments for type 2 diabetes mellitus (T2DM) and obesity, achieving unprecedented weight loss and cardiometabolic benefits [2]. The global market for GLP-1 RAs has expanded exponentially, with agents such as semaglutide (Ozempic^®^, Wegovy^®^), liraglutide (Victoza^®^, Saxenda^®^), tirzepatide (Mounjaro^®^), and exenatide (Byetta^®^) now widely prescribed for metabolic disease management [3].

The intersection of incretin therapy and athletic performance has garnered attention due to several pharmacological properties that could theoretically enhance competitive outcomes. These include profound effects on body composition, particularly in weight-category sports where rapid and sustained fat loss is advantageous; potential improvements in cardiovascular function and exercise capacity; modulation of energy metabolism and substrate utilization; and anti-inflammatory effects that may accelerate recovery [4,5]. Recent preclinical studies have demonstrated that GLP-1 receptor activation enhances exercise endurance, promotes skeletal muscle remodeling toward oxidative phenotypes, and augments mitochondrial biogenesis through AMPK-dependent pathways [6].

The World Anti-Doping Agency (WADA) maintains a Prohibited List that categorizes substances and methods banned in competitive sports based on three criteria: (1) potential to enhance performance, (2) actual or potential health risks, and (3) violation of the spirit of sport [7]. The list is organized into categories including anabolic agents (S1), peptide hormones and growth factors (S2), beta-2 agonists (S3), hormone and metabolic modulators (S4), and diuretics and masking agents (S5) [8]. As of 2026, incretin-based therapies are not explicitly listed on the WADA Prohibited List, yet their growing use in non-diabetic populations for weight management and aesthetic purposes raises questions about potential misuse in athletic contexts [9].

The ethical and regulatory considerations surrounding incretin therapy in sports are complex. Unlike traditional performance-enhancing drugs such as anabolic steroids or erythropoietin, incretin therapies are prescribed for legitimate medical conditions and may be used by athletes with T2DM under Therapeutic Use Exemptions (TUEs) [7]. However, the distinction between therapeutic use and performance enhancement becomes blurred when these agents are employed primarily for body composition manipulation in weight-sensitive sports such as wrestling, boxing, mixed martial arts, and aesthetic disciplines like gymnastics and figure skating [7].

This narrative review aims to comprehensively evaluate the available evidence regarding incretin-based therapies and their potential classification as doping agents. We examine the molecular mechanisms of incretin hormones and their pharmacological agents, metabolic effects relevant to athletic performance, potential performance-enhancing effects in various sporting contexts, current WADA anti-doping regulations, and ethical considerations. By synthesizing data from preclinical studies, clinical trials, pharmacovigilance reports, and regulatory frameworks, we seek to provide an evidence-based assessment of whether incretin therapy should be considered doping in sports.

## 2. Methods

This narrative review was conducted following a systematic literature search. PubMed, Scopus, and Web of Science databases were searched without date restrictions using the following search terms: “GLP-1 receptor agonists”, “glucagon-like peptide-1”, “DPP-4 inhibitors”, “dipeptidyl peptidase-4”, “incretin therapy”, “doping”, “athletic performance”, “WADA”, “sports pharmacology”, “body composition”, “exercise capacity”, and “anti-doping”. Reference lists of relevant articles were also screened for additional sources. Preclinical studies, clinical trials, pharmacovigilance reports, and regulatory documents were included. The graphical abstract was created using BioRender (BioRender.com).

## 3. Incretin Hormones and Their Pharmacological Agents

### 3.1. Physiology of Incretin Hormones

The incretin effect refers to the phenomenon whereby oral glucose administration elicits a greater insulin secretory response compared to intravenous glucose delivery, accounting for 50–70% of postprandial insulin secretion in healthy individuals [3]. This effect is mediated primarily by two gut-derived peptide hormones: glucagon-like peptide-1 (GLP-1) and glucose-dependent insulinotropic polypeptide (GIP), both secreted by enteroendocrine cells in response to nutrient ingestion [3]. GLP-1 is produced by L-cells in the distal ileum and colon through post-translational processing of proglucagon, while GIP is secreted by K-cells in the proximal small intestine [3].

GLP-1 exerts its effects through binding to the GLP-1 receptor (GLP-1R), a G-protein-coupled receptor expressed in pancreatic beta cells, central nervous system, cardiovascular tissues, skeletal muscle, and adipose tissue [6]. Receptor activation stimulates adenylyl cyclase, increasing intracellular cyclic AMP (cAMP) levels and activating protein kinase A (PKA) and exchange protein directly activated by cAMP (EPAC) pathways [3]. In pancreatic beta cells, this cascade enhances glucose-dependent insulin secretion, suppresses glucagon release, and promotes beta-cell proliferation and survival [3]. Beyond glycemic control, GLP-1R activation in the central nervous system reduces appetite and food intake through hypothalamic and brainstem circuits, contributing to weight loss [2].

GIP, acting through the GIP receptor (GIPR), similarly enhances glucose-dependent insulin secretion but exhibits distinct tissue distribution and metabolic effects [10]. Notably, GLP-1R is predominantly expressed in pancreatic beta cells, the central nervous system, gastrointestinal tract, heart, kidney, and lung, with limited expression in skeletal muscle and adipose tissue. In contrast, GIPR shows broader peripheral expression, including high levels in adipose tissue, bone, adrenal cortex, pituitary, and skeletal muscle, as well as central nervous system regions. This differential receptor distribution underlies the distinct metabolic effects of the two incretins: while GLP-1 predominantly mediates satiety and gastric emptying inhibition, GIP exerts stronger anabolic effects on adipose tissue and bone metabolism and may play a more direct role in skeletal muscle substrate utilization. Recent structural studies have elucidated the molecular basis for dual and triple agonism at GLP-1R, GIPR, and glucagon receptors, revealing conformational dynamics that enable selective or combined receptor activation [10]. This mechanistic understanding has facilitated the development of next-generation co-agonists with enhanced metabolic efficacy.

### 3.2. GLP-1 Receptor Agonists: Mechanisms of Action

GLP-1 RAs are synthetic peptides or modified analogs designed to resist degradation by dipeptidyl peptidase-4 (DPP-4), the enzyme responsible for rapid inactivation of native GLP-1 (half-life ~2 min) [3]. These agents are classified by duration of action: short-acting (exenatide twice daily), intermediate-acting (liraglutide once daily), and long-acting (semaglutide once weekly, dulaglutide once weekly) [3]. At the molecular level, GLP-1 RAs bind to GLP-1R with high affinity, triggering conformational changes that stabilize the active receptor state and prolong intracellular signaling [11].

The downstream signaling cascades activated by GLP-1R include the cAMP/PKA pathway, which phosphorylates transcription factors such as cAMP response element-binding protein (CREB), enhancing expression of genes involved in insulin biosynthesis, beta-cell survival, and glucose metabolism [3]. Additionally, GLP-1R activation stimulates the phosphatidylinositol 3-kinase (PI3K)/Akt pathway, promoting glucose transporter 4 (GLUT4) translocation and glucose uptake in skeletal muscle and adipose tissue [6]. In skeletal muscle, GLP-1R signaling activates AMP-activated protein kinase (AMPK), a master regulator of cellular energy homeostasis that enhances mitochondrial biogenesis, fatty acid oxidation, and oxidative fiber-type transformation [6].

Semaglutide, a modified GLP-1 analog with 94% homology to native GLP-1, incorporates an amino acid substitution and fatty acid side chain that enable albumin binding and prolonged half-life (~7 days) [2]. Clinical trials demonstrate semaglutide induces 15–20% body weight reduction at 2.4 mg weekly dosing, with preferential loss of visceral adipose tissue [2]. Liraglutide, a once-daily GLP-1 RA with 97% homology to human GLP-1, achieves 5–10% weight loss through central appetite suppression and peripheral metabolic effects [5]. Tirzepatide, a dual GIP/GLP-1 receptor agonist, represents a novel therapeutic class that harnesses synergistic incretin signaling to achieve superior glycemic control and weight loss (up to 22.5% body weight reduction) compared to selective GLP-1 RAs [2].

### 3.3. DPP-4 Inhibitors: Mechanisms of Action

DPP-4 inhibitors (gliptins) constitute an alternative incretin-based strategy that enhances endogenous GLP-1 and GIP activity by preventing their enzymatic degradation [3]. Agents such as sitagliptin, vildagliptin, saxagliptin, and linagliptin competitively inhibit DPP-4, a serine protease expressed on endothelial cells, immune cells, and in soluble form in plasma [12]. By prolonging the half-life of active incretins from minutes to hours, DPP-4 inhibitors augment postprandial insulin secretion and suppress glucagon release in a glucose-dependent manner [3].

At the molecular level, DPP-4 inhibitors bind to the enzyme’s active site, forming reversible or slowly reversible complexes that prevent substrate cleavage [12]. Unlike GLP-1 RAs, DPP-4 inhibitors do not achieve supraphysiological incretin levels and consequently exhibit more modest glycemic efficacy and weight-neutral effects [3]. The absence of significant weight loss with DPP-4 inhibitors reflects their inability to activate central GLP-1R pathways at concentrations sufficient to suppress appetite [3]. However, DPP-4 inhibitors demonstrate favorable safety profiles with low hypoglycemia risk and oral bioavailability, making them attractive first-line agents for T2DM management [12].

### 3.4. Tissue-Specific Actions and Pleiotropic Effects

Beyond pancreatic and central nervous system effects, incretin hormones exert pleiotropic actions across multiple organ systems relevant to athletic performance. In the cardiovascular system, GLP-1R activation improves endothelial function, reduces blood pressure, and enhances myocardial contractility through nitric oxide-dependent mechanisms [13]. Cardiovascular outcome trials have demonstrated that GLP-1 RAs reduce major adverse cardiovascular events in patients with T2DM and established cardiovascular disease, effects attributed to anti-inflammatory, anti-atherosclerotic, and cardioprotective signaling [13].

In skeletal muscle, GLP-1R expression and signaling modulate glucose uptake, glycogen synthesis, and mitochondrial function [6]. Preclinical studies demonstrate that GLP-1 overexpression or exendin-4 (a GLP-1R agonist) treatment enhances endurance capacity, increases type I oxidative fiber proportion, and augments mitochondrial biogenesis through AMPK-dependent pathways [6]. These effects suggest potential performance-enhancing properties in endurance sports, although translation to human athletes remains uncertain.

In adipose tissue, GLP-1R activation promotes lipolysis, thermogenesis, and browning of white adipose tissue, contributing to fat mass reduction [2]. GLP-1 RAs preferentially reduce visceral and ectopic fat depots, which are metabolically deleterious and associated with insulin resistance [2]. However, weight loss induced by GLP-1 RAs is accompanied by proportional reductions in lean mass (20–30% of total weight loss), raising concerns about sarcopenia and functional impairment, particularly in older adults and athletes [2].

Anti-inflammatory effects of GLP-1R activation have been documented in multiple tissues, including brain, vasculature, and immune cells [14]. GLP-1 RAs suppress pro-inflammatory cytokine production (TNF-α, IL-6, IL-1β), inhibit NF-κB signaling, and promote M2 macrophage polarization [14]. These anti-inflammatory properties may accelerate recovery from exercise-induced muscle damage and reduce chronic low-grade inflammation associated with obesity and metabolic syndrome [15].

## 4. Metabolic Effects Relevant to Athletic Performance

### 4.1. Body Composition and Weight Management

Body composition manipulation is a critical determinant of athletic performance across numerous sports, particularly weight-category disciplines (wrestling, boxing, judo, weightlifting) and aesthetic sports (gymnastics, figure skating, diving) where power-to-weight ratio and visual presentation are paramount [1]. GLP-1 RAs induce substantial and sustained weight loss through complementary mechanisms: central appetite suppression via hypothalamic GLP-1R activation, delayed gastric emptying that prolongs satiety, and peripheral metabolic effects that enhance energy expenditure [2].

Clinical trials demonstrate that semaglutide 2.4 mg weekly achieves 15–20% body weight reduction over 68 weeks in individuals with obesity, with 86% of participants achieving ≥5% weight loss and 69% achieving ≥10% weight loss [2]. Liraglutide 3.0 mg daily produces 5–10% weight loss, while tirzepatide 15 mg weekly achieves up to 22.5% weight reduction, surpassing all currently available pharmacotherapies [2]. Importantly, weight loss with GLP-1 RAs is predominantly from fat mass, with preferential reduction in visceral adipose tissue and ectopic fat depots (liver, pancreas, myocardium) [2].

However, a critical limitation of GLP-1 RA-induced weight loss is the concurrent reduction in lean mass, which comprises 20–30% of total weight loss [2]. This proportional lean mass loss is comparable to or slightly higher than that observed with caloric restriction alone, raising concerns about preservation of muscle mass and functional capacity [2]. In athletes, loss of lean mass could impair strength, power output, and metabolic capacity, potentially offsetting benefits of fat mass reduction [16]. The extent to which lean mass loss translates into functional impairment remains unclear, as most studies have not assessed muscle strength, power, or sport-specific performance outcomes [2].

Strategies to mitigate lean mass loss during GLP-1 RA therapy include resistance training, adequate protein intake (1.6–2.2 g/kg/day), and potentially anabolic co-interventions [10]. One study combining liraglutide with exercise training in diet-induced obesity demonstrated synergistic effects on vascular inflammation and metabolic insulin action, suggesting that structured exercise programs may optimize body composition outcomes during incretin therapy [15]. However, data on resistance training combined with GLP-1 RAs in athletes or physically active individuals are lacking.

### 4.2. Energy Metabolism and Substrate Utilization

Energy metabolism and substrate utilization are fundamental determinants of athletic performance, particularly in endurance sports where efficient oxidation of carbohydrates and fats sustains prolonged exercise [1]. GLP-1R activation influences multiple aspects of energy metabolism, including glucose uptake, glycogen synthesis, fatty acid oxidation, and mitochondrial function [6].

In skeletal muscle, GLP-1R signaling enhances glucose uptake through AMPK-dependent GLUT4 translocation, increasing substrate availability for glycolysis and oxidative phosphorylation [6]. Preclinical studies demonstrate that GLP-1 overexpression or exendin-4 treatment augments glycogen synthesis in skeletal muscle, potentially enhancing glycogen stores available for high-intensity exercise [6]. Additionally, GLP-1R activation promotes mitochondrial biogenesis through upregulation of peroxisome proliferator-activated receptor gamma coactivator 1-alpha (PGC-1α), a master regulator of mitochondrial gene expression [6].

Complementary preclinical evidence underscores the direct effects of GLP-1R agonists on intramuscular lipid handling independently of weight loss. Xu et al. demonstrated that short-term exenatide treatment significantly reduced intramyocellular lipid accumulation in obese (ob/ob) and diet-induced obese mice through AMPK activation and upregulation of lipid-oxidation enzymes; concordant results were obtained in palmitate-loaded C2C12 myotubes, establishing a direct myocellular action of GLP-1R agonism distinct from systemic body weight effects [17]. Extending these observations, Xiang et al. reported that both liraglutide and semaglutide improved high-fat-diet-induced skeletal muscle atrophy in mice by restoring GLUT4 and SIRT1 expression; pharmacological inhibition of SIRT1 attenuated these metabolic benefits, implicating the SIRT1 deacetylase pathway as an additional mechanistic node governing GLP-1-mediated muscle glucose handling and protein homeostasis [18].

The shift toward oxidative metabolism induced by GLP-1R signaling is evidenced by increased type I fiber proportion and enhanced mitochondrial respiration in skeletal muscle [6]. Type I fibers, characterized by high oxidative capacity and fatigue resistance, are advantageous for endurance performance [6]. However, the relevance of these preclinical findings to human athletes is uncertain, as most studies have been conducted in rodent models with supraphysiological GLP-1 levels or genetic overexpression [6].

Recent in vivo studies provide more granular mechanistic insight into mitochondrial adaptations. Semaglutide administration in a murine model of sarcopenic obesity increased mitochondrial number and cross-sectional area in the gastrocnemius muscle and elevated the type I-to-type II fiber ratio, consistent with a shift toward an oxidative metabolic phenotype [19]. Building on these morphological findings, Choi et al. demonstrated that semaglutide-induced weight loss in diet-induced obese mice improved skeletal muscle mitochondrial energy efficiency, specifically increasing the ATP/O2 coupling ratio (P/O) in permeabilized fiber preparations and inducing proteomic remodeling of Complex III assembly factors without major changes in OXPHOS subunit abundance, suggesting qualitative improvement in electron transport chain function [20]. A systematic review by Old et al. synthesizing available preclinical and early clinical data confirmed that GLP-1R agonists consistently increase mitochondrial area and number in skeletal muscle across multiple experimental models; however, effects on respiratory capacity, uncoupling proteins, and PGC-1α expression showed greater inter-study variability, highlighting the need for standardized outcome measures and dose-matched comparisons [21].

Fatty acid oxidation is enhanced by GLP-1R activation through AMPK-mediated phosphorylation and inhibition of acetyl-CoA carboxylase (ACC), reducing malonyl-CoA levels and relieving inhibition of carnitine palmitoyltransferase 1 (CPT1), the rate-limiting enzyme for mitochondrial fatty acid uptake [6]. Increased reliance on fat oxidation during submaximal exercise could spare glycogen and delay fatigue in endurance events [1]. However, clinical studies in humans have not consistently demonstrated enhanced fat oxidation or improved exercise performance with GLP-1 RA therapy [5].

In addition to AMPK-mediated CPT1 disinhibition, GLP-1R agonism may engage the PGC-1α/irisin axis to coordinate muscle–adipose metabolic crosstalk. Zhang et al. showed that liraglutide increased PGC-1α and FNDC5 expression in C2C12 myotubes exposed to palmitic acid, promoting irisin secretion into the conditioned medium; in vivo, this was associated with enhanced UCP1 expression in white adipose tissue, suggesting that GLP-1R activation in skeletal muscle contributes to thermogenic remodeling through an endocrine mechanism [22]. While the direct relevance of the muscle–adipose irisin axis to exercise performance in athletes remains to be established, it provides an additional pathway through which incretin-based therapies may modulate whole-body energy expenditure and substrate oxidation beyond direct myocellular AMPK activation.

### 4.3. Cardiovascular Function and Cardioprotection

Cardiovascular function is a critical determinant of aerobic capacity and endurance performance, with maximal oxygen uptake (VO2max) serving as the gold standard measure of cardiorespiratory fitness [1]. GLP-1R activation exerts multiple cardiovascular effects that could theoretically enhance athletic performance, including improved endothelial function, reduced blood pressure, enhanced myocardial contractility, and cardioprotection against ischemia–reperfusion injury [13].

GLP-1 RAs improve endothelial function through nitric oxide (NO)-dependent mechanisms, enhancing vasodilation and blood flow to exercising muscles [13]. Improved endothelial function is associated with increased exercise capacity in patients with cardiovascular disease, although effects in healthy athletes are unknown [13]. GLP-1 RAs also reduce systolic and diastolic blood pressure by 2–5 mmHg, effects attributed to natriuresis, vasodilation, and sympathetic modulation [13]. While modest, these blood pressure reductions could improve cardiovascular efficiency during exercise.

Cardiovascular outcome trials have demonstrated that GLP-1 RAs reduce major adverse cardiovascular events (MACEs) by 12–26% in patients with T2DM and established cardiovascular disease [13]. Mechanisms underlying cardioprotection include anti-inflammatory effects, reduced atherosclerotic plaque progression, improved lipid profiles, and direct myocardial effects [13]. GLP-1R activation in cardiomyocytes enhances glucose uptake, reduces apoptosis, and improves contractility through cAMP/PKA and PI3K/Akt signaling [13].

However, clinical trials evaluating GLP-1 RAs in athletes or healthy individuals have not demonstrated consistent improvements in VO2max, exercise capacity, or endurance performance [5]. One randomized controlled trial (LIPER2) assessed the effect of liraglutide on physical performance in patients with T2DM, finding no significant improvement in VO2max, 6-min walk distance, or muscle strength compared to placebo [5]. These findings suggest that cardiovascular benefits of GLP-1 RAs may be most pronounced in individuals with pre-existing cardiovascular disease or metabolic dysfunction, rather than healthy athletes with normal cardiovascular function.

### 4.4. Anti-Inflammatory Effects and Recovery

Inflammation plays a dual role in athletic performance: acute exercise-induced inflammation is necessary for adaptive responses and muscle remodeling, while chronic low-grade inflammation impairs recovery and performance [15]. GLP-1R activation exerts potent anti-inflammatory effects across multiple tissues, suppressing pro-inflammatory cytokine production and promoting resolution of inflammation [14].

At the molecular level, GLP-1R signaling inhibits nuclear factor-kappa B (NF-κB) activation, a master regulator of inflammatory gene expression [14]. GLP-1 RAs reduce circulating levels of tumor necrosis factor-alpha (TNF-α), interleukin-6 (IL-6), and C-reactive protein (CRP) in patients with obesity and T2DM [14]. These anti-inflammatory effects are mediated through direct GLP-1R activation in immune cells (macrophages, T cells) and indirect effects via weight loss and improved metabolic health [14].

In the context of athletic performance, anti-inflammatory effects of GLP-1 RAs could theoretically accelerate recovery from exercise-induced muscle damage and reduce delayed-onset muscle soreness (DOMS) [15]. One study combining liraglutide with exercise training demonstrated synergistic attenuation of vascular inflammation and enhanced metabolic insulin action in early diet-induced obesity [15]. However, whether these anti-inflammatory effects translate to improved recovery or performance in athletes remains speculative, as no studies have directly assessed recovery outcomes following GLP-1 RA administration in athletic populations.

Importantly, excessive suppression of exercise-induced inflammation could potentially impair adaptive responses to training, as inflammatory signaling is necessary for muscle protein synthesis, satellite cell activation, and mitochondrial biogenesis [10]. The optimal balance between anti-inflammatory effects and preservation of adaptive responses is unknown and may vary depending on training status, exercise modality, and individual characteristics [10].

## 5. Potential Performance-Enhancing Effects in Athletes

### 5.1. Endurance Sports

Endurance sports (marathon running, cycling, triathlon, cross-country skiing) demand sustained aerobic metabolism, efficient substrate utilization, and resistance to fatigue over prolonged durations [1]. Theoretical performance-enhancing effects of incretin therapy in endurance athletes include enhanced mitochondrial biogenesis, increased oxidative fiber proportion, improved glucose uptake and glycogen synthesis, and optimized body composition through fat mass reduction [6].

Preclinical evidence provides the strongest support for endurance-enhancing effects of GLP-1R activation. Wu et al. demonstrated that GLP-1 overexpression in skeletal muscle enhanced endurance capacity and promoted remodeling toward oxidative (type I) fiber phenotypes via GLP-1R/AMPK signaling [6]. These findings were corroborated by studies showing that exendin-4 administration in rodents increased mitochondrial biogenesis markers (PGC-1alpha, TFAM) and improved maximal oxygen consumption. Further preclinical work has demonstrated that GLP-1R agonists attenuate skeletal muscle atrophy in models of disuse and aging, preserving muscle mass and contractile function through PI3K/Akt and mTOR-dependent pathways [3]. In adipose tissue, GLP-1R activation promotes lipolysis and reduces lipid droplet accumulation, while in cardiac muscle, GLP-1R signaling improves contractile efficiency and reduces ischemia–reperfusion injury. Animal models of diet-induced obesity have shown that liraglutide combined with exercise synergistically attenuates vascular inflammation and enhances insulin-mediated glucose uptake [6]. Additionally, GLP-1R agonists have demonstrated anti-inflammatory effects in the central nervous system, potentially relevant to exercise-induced neuroinflammation and recovery [3]. However, translation of these preclinical findings to human athletes is highly uncertain. Clinical trials in humans have not demonstrated consistent improvements in exercise capacity or endurance performance, and most studies have been conducted in patients with T2DM or obesity rather than healthy athletes [5]. Two additional preclinical studies published in 2025 and 2026 provide important mechanistic counterbalance to these endurance-enhancing observations. Jeromson et al. demonstrated that semaglutide-treated diet-induced obese mice exhibited reductions in skeletal muscle mass and contractile strength comparable in magnitude to those achieved by matched caloric restriction alone, and that both muscle mass and function recovered following treatment withdrawal; these findings indicate that functional impairment may be substantially attributable to caloric deficit rather than a direct pharmacological effect on muscle biology [23]. Conversely, Abuetabh et al. reported that semaglutide-treated obese mice showed significant reductions in lean mass, muscle endurance, and peak strength accompanied by suppression of mitochondrial gene expression; crucially, co-administration of a ketone ester supplement blunted these functional deficits and partially preserved mitochondrial transcriptional programs, suggesting that alternative energy substrates can mitigate GLP-1 RA-related adverse effects on muscle bioenergetics [24]. Collectively, these studies underscore that the net impact of GLP-1 RA therapy on preclinical exercise performance is highly contingent on energy balance, concurrent nutritional strategies, and the metabolic phenotype of the experimental subject.

However, translation of these preclinical findings to human athletes is highly uncertain. Clinical trials in humans have not demonstrated consistent improvements in exercise capacity or endurance performance with GLP-1 RA therapy [5]. The LIPER2 trial found no significant effect of liraglutide on VO2max, 6-min walk distance, or muscle strength in patients with T2DM [5]. Importantly, this study was conducted in individuals with metabolic disease rather than trained athletes, and the 26-week intervention may have been insufficient to detect performance adaptations [5].

A critical consideration is that endurance athletes typically maintain low body fat percentages (5–15%) and high lean mass, contrasting with the obesity populations in which GLP-1 RAs are studied [1]. The substantial weight loss induced by GLP-1 RAs, including 20–30% lean mass loss, could be detrimental to endurance athletes who rely on muscle mass for power output and metabolic capacity [2]. Furthermore, appetite suppression and delayed gastric emptying may impair athletes’ ability to consume adequate calories and carbohydrates to support high training volumes [25].

### 5.2. Strength and Power Sports

Strength and power sports (weightlifting, powerlifting, sprinting, throwing events) prioritize maximal force production, explosive power, and muscle mass [1]. The potential performance-enhancing effects of incretin therapy in these disciplines are limited and potentially detrimental due to lean mass loss accompanying weight reduction [16].

GLP-1 RAs induce absolute reductions in lean mass comprising 20–30% of total weight loss, which could impair maximal strength and power output [2]. Muscle mass is a primary determinant of force-generating capacity, and loss of contractile tissue would be expected to reduce performance in strength-dependent events [16]. Additionally, appetite suppression and reduced caloric intake may compromise athletes’ ability to maintain positive energy balance necessary for muscle hypertrophy and strength gains [25].

No clinical trials have evaluated the effects of GLP-1 RAs on strength, power, or muscle hypertrophy in athletes or resistance-trained individuals. The LIPER2 trial found no significant effect of liraglutide on handgrip strength or leg extension strength in patients with T2DM, although these measures may not adequately reflect sport-specific strength performance [5]. Importantly, participants in this trial were not engaged in structured resistance training, which could modulate the effects of GLP-1 RAs on muscle mass and function [5].

Theoretical strategies to mitigate lean mass loss during GLP-1 RA therapy include high-protein diets (≥2.0 g/kg/day), progressive resistance training, and potentially anabolic co-interventions [10]. However, the efficacy of these strategies in preserving muscle mass during GLP-1 RA-induced weight loss in athletes has not been systematically evaluated [10]. Given the current evidence, incretin therapy appears unlikely to enhance performance in strength and power sports and may be detrimental if lean mass loss is not adequately mitigated [16].

### 5.3. Weight-Category Sports and Body Weight Management

Weight-category sports (wrestling, boxing, judo, weightlifting, rowing) require athletes to compete within specific weight classes, creating strong incentives for rapid and sustained weight loss to gain competitive advantages against smaller opponents [1]. Traditional weight-cutting practices involve severe caloric restriction, dehydration, and diuretic use in the days preceding weigh-in, followed by rapid rehydration and refeeding [1]. These practices are associated with health risks including dehydration, electrolyte imbalances, impaired cognitive function, and increased injury risk [1].

GLP-1 RAs offer a pharmacological approach to sustained weight loss that could theoretically provide competitive advantages in weight-category sports [2]. The substantial and sustained weight loss achieved with semaglutide (15–20%), liraglutide (5–10%), and tirzepatide (up to 22.5%) could enable athletes to compete in lower weight classes while maintaining or minimizing loss of lean mass and functional capacity [2]. Unlike acute dehydration-based weight cutting, GLP-1 RA-induced weight loss occurs gradually over weeks to months, potentially allowing physiological adaptation and preservation of performance [2].

However, several factors limit the applicability of GLP-1 RAs for weight management in athletes. First, the 20–30% lean mass loss accompanying weight reduction could impair strength, power, and metabolic capacity, offsetting benefits of competing in a lower weight class [2]. Second, appetite suppression and delayed gastric emptying may impair athletes’ ability to rapidly refuel following weigh-in, a critical component of weight-cutting strategies [25]. Third, gastrointestinal side effects (nausea, vomiting, diarrhea) are common with GLP-1 RAs and could impair training and competition performance [2].

Importantly, the use of GLP-1 RAs for weight management in non-diabetic athletes would constitute off-label use and raise ethical concerns about medicalization of weight cutting and potential health risks [9]. Pharmacovigilance data have documented cases of semaglutide misuse for cosmetic weight loss, including adverse events such as severe gastrointestinal symptoms, gallbladder disease, and pancreatitis [9]. The risk–benefit profile of GLP-1 RAs in healthy athletes seeking weight loss for competitive advantage remains undefined and warrants careful consideration [9].

### 5.4. Aesthetic and Judged Sports

Aesthetic and judged sports (gymnastics, figure skating, diving, bodybuilding, physique competitions) emphasize visual presentation, body composition, and subjective evaluation of performance [1]. In these disciplines, low body fat percentage and favorable muscle-to-fat ratio are critical determinants of competitive success [1]. GLP-1 RAs could theoretically provide performance advantages through preferential reduction in fat mass, particularly visceral and subcutaneous adipose tissue, while preserving relative muscle definition [2].

Bodybuilding and physique competitions explicitly prioritize muscle mass, symmetry, and leanness, with athletes typically achieving body fat percentages of 5–10% for men and 10–15% for women during competition [1]. Traditional approaches to contest preparation involve severe caloric restriction, high-protein diets, and often illicit use of anabolic steroids, thyroid hormones, and diuretics [1]. GLP-1 RAs could offer an alternative or adjunctive approach to fat loss, although the concurrent lean mass loss would be highly undesirable in these disciplines [16].

Anecdotal reports and social media discussions suggest emerging use of GLP-1 RAs among bodybuilders and fitness enthusiasts for contest preparation and aesthetic enhancement [9]. However, no systematic data exist on the prevalence, patterns, or outcomes of GLP-1 RA use in these populations [9]. The substantial lean mass loss (20–30% of total weight loss) would be expected to impair competitive outcomes in bodybuilding, where muscle mass is the primary judged criterion [2].

In gymnastics, figure skating, and diving, where power-to-weight ratio and aesthetic presentation are both important, the effects of GLP-1 RAs are more ambiguous [1]. Reduction in body weight could enhance power-to-weight ratio and facilitate execution of complex aerial maneuvers, while fat loss could improve aesthetic presentation [1]. However, loss of lean mass could impair strength and power necessary for explosive movements, and gastrointestinal side effects could interfere with training and competition [2].

## 6. WADA Anti-Doping Regulations and Incretin Therapy

### 6.1. WADA Prohibited List: Structure and Criteria

The World Anti-Doping Agency (WADA) maintains the Prohibited List, a comprehensive catalog of substances and methods banned in competitive sports, updated annually through a rigorous consultation process involving scientific experts, athletes, and international federations [7]. Substances are classified as prohibited at all times (in- and out-of-competition), prohibited in-competition only, or prohibited in particular sports [7]. The list is organized into categories: S0 (non-approved substances), S1 (anabolic agents), S2 (peptide hormones, growth factors, and related substances), S3 (beta-2 agonists), S4 (hormone and metabolic modulators), S5 (diuretics and masking agents), and methods (M1-M3) [7].

Inclusion of a substance on the Prohibited List requires satisfaction of at least two of three criteria: (1) the substance has the potential to enhance or enhances sport performance; (2) the use of the substance represents an actual or potential health risk to the athlete; and (3) the use of the substance violates the spirit of sport [7]. The “spirit of sport” encompasses values such as ethics, fair play, honesty, health, excellence in performance, character and education, fun and joy, teamwork, dedication and commitment, respect for rules and laws, respect for self and other participants, courage, and community and solidarity [7].

WADA’s scientific review process evaluates emerging substances based on pharmacological mechanisms, preclinical and clinical evidence of performance enhancement, health risks, prevalence of use in athletic populations, and detectability in anti-doping testing [8]. Substances demonstrating clear performance-enhancing effects with established detection methods are prioritized for inclusion, while substances with theoretical but unproven effects may be monitored without prohibition [8].

### 6.2. Current Status of Incretin Therapies on the Prohibited List

As of the 2026 WADA Prohibited List, incretin-based therapies including GLP-1 receptor agonists (semaglutide, liraglutide, tirzepatide, exenatide, dulaglutide) and DPP-4 inhibitors (sitagliptin, vildagliptin, saxagliptin, linagliptin) are not explicitly listed as prohibited substances [7]. This regulatory status reflects the current assessment that incretin therapies do not meet the criteria for inclusion based on available evidence of performance enhancement, health risks, and prevalence of misuse in athletic populations [8].

However, several considerations suggest that incretin therapies warrant continued surveillance and potential future evaluation for inclusion on the Prohibited List. First, the rapid expansion of GLP-1 RA use for obesity management in non-diabetic populations has increased accessibility and awareness of these agents among athletes and the general public [9]. Second, emerging evidence of misuse for cosmetic weight loss and body composition manipulation raises concerns about off-label use in athletic contexts [9]. Third, preclinical studies demonstrating endurance-enhancing effects and metabolic adaptations suggest theoretical performance-enhancing potential that may become clinically relevant as use patterns evolve [6].

The absence of incretin therapies from the Prohibited List does not preclude their potential classification under existing categories. For example, if future evidence demonstrates that GLP-1 RAs function as metabolic modulators with clear performance-enhancing effects, they could be included under category S4 (hormone and metabolic modulators), which encompasses substances that alter hormonal and metabolic pathways [7]. Alternatively, if incretin therapies are found to be used primarily as masking agents or to manipulate body weight for competitive advantage, they could be considered under S5 (diuretics and masking agents) [7].

### 6.3. Evaluation Against WADA Criteria

To assess whether incretin therapies should be considered doping, we evaluate them against the three WADA criteria for inclusion on the Prohibited List:

Criterion 1: Potential to Enhance Sport Performance

The evidence for performance-enhancing effects of incretin therapies is mixed and context-dependent. Preclinical studies demonstrate that GLP-1R activation enhances endurance capacity, promotes oxidative muscle fiber transformation, and augments mitochondrial biogenesis through AMPK-dependent mechanisms [6]. These effects suggest potential benefits for endurance sports. However, clinical trials in humans have not consistently demonstrated improvements in exercise capacity, VO2max, or functional performance [5].

In weight-category sports, the substantial weight loss induced by GLP-1 RAs (15–22.5% body weight) could provide competitive advantages by enabling athletes to compete in lower weight classes [2]. However, the concurrent loss of 20–30% lean mass could impair strength, power, and metabolic capacity, potentially offsetting benefits [2]. In strength and power sports, lean mass loss would be expected to impair performance [16].

Overall, the performance-enhancing potential of incretin therapies appears limited and highly context-dependent, with theoretical benefits in specific scenarios (endurance sports, weight-category sports) but lack of consistent evidence in human athletes [5]. This criterion is partially met but with substantial uncertainty.

Criterion 2: Actual or Potential Health Risk

GLP-1 RAs are approved therapeutic agents with established safety profiles in patients with T2DM and obesity [2]. Common adverse effects include gastrointestinal symptoms (nausea, vomiting, diarrhea, constipation), which occur in 30–50% of users but typically diminish over time [2]. Serious adverse effects are rare but include pancreatitis, gallbladder disease, acute kidney injury, and potential thyroid C-cell tumors (based on rodent studies) [2].

In the context of athletic use, potential health risks include:

- Excessive weight loss and malnutrition if used by individuals with normal or low body weight [9]
- Lean mass loss and potential sarcopenia, particularly if not combined with resistance training and adequate protein intake [2]
- Gastrointestinal side effects that could impair training, competition performance, and nutritional intake [2]
- Hypoglycemia risk if combined with other glucose-lowering agents or during prolonged exercise [3]
- Unknown long-term effects of use in healthy, non-diabetic athletes [9]. These safety concerns are particularly relevant in the athletic context: the main adverse effects of GLP-1 RAs include gastrointestinal symptoms (nausea, vomiting, diarrhea) occurring in up to 40% of users, as well as potential risks of pancreatitis, gallbladder disease, and thyroid C-cell tumors observed in rodent models. Chronic use in athletes may pose additional risks: the significant reduction in lean body mass (20–30% of total weight loss) could impair strength, power output, and injury resilience. Furthermore, appetite-suppressive effects may contribute to relative energy deficiency in sport (RED-S), a syndrome associated with hormonal disruption, bone stress injuries, and immune suppression. Athletes considering these agents for weight management should be counseled that long-term safety data in healthy, non-diabetic individuals remain unavailable, and that the risk–benefit profile in this population differs fundamentally from that in patients with T2DM or obesity.

Pharmacovigilance data have documented cases of semaglutide misuse for cosmetic weight loss, including adverse events such as severe gastrointestinal symptoms, dehydration, and electrolyte imbalances [9]. However, the overall health risk profile of GLP-1 RAs in healthy athletes remains poorly characterized due to a lack of systematic data [9].

This criterion is partially met, with established risks in therapeutic populations but uncertain risk profile in healthy athletes using these agents for performance enhancement or weight management [9].

Criterion 3: Violation of the Spirit of Sport

The “spirit of sport” criterion is inherently subjective and reflects ethical values including fair play, honesty, health, and respect for rules [7]. Several considerations are relevant to evaluating incretin therapies against this criterion:

- Medicalization of performance enhancement: Use of prescription medications for competitive advantage rather than medical necessity could be viewed as violating principles of fair play and natural athletic ability [1]
- Accessibility and equity: GLP-1 RAs are expensive (often $1000–1500 per month) and may not be accessible to all athletes, creating potential inequities based on financial resources [2]
- Therapeutic use vs. enhancement: Athletes with legitimate medical conditions (T2DM, obesity) may require incretin therapy, complicating distinctions between therapeutic use and performance enhancement [7]
- Precedent and consistency: Many approved therapeutic agents (e.g., beta-2 agonists, corticosteroids) are permitted with Therapeutic Use Exemptions, suggesting that therapeutic status alone does not preclude athletic use [7]

The spirit of sport criterion is ambiguous for incretin therapies, as reasonable arguments can be made both for and against classification as violations of sporting ethics [1].

### 6.4. Therapeutic Use Exemptions (TUE)

Athletes with diagnosed medical conditions requiring prohibited substances may apply for Therapeutic Use Exemptions (TUEs), which permit use of otherwise banned substances under specific conditions [7]. TUE criteria require that: (1) the athlete would experience significant health impairment without the substance; (2) therapeutic use would not produce significant enhancement beyond return to normal health; (3) no reasonable permitted therapeutic alternative exists; and (4) the need for the substance is not a consequence of prior non-therapeutic use [7].

For athletes with T2DM or obesity meeting clinical criteria for incretin therapy, TUEs could theoretically be granted [7]. However, several considerations complicate TUE applications for incretin therapies:

- Therapeutic alternatives: Multiple alternative treatments exist for T2DM (metformin, sulfonylureas, insulin) and obesity (lifestyle modification, other pharmacotherapies), potentially negating the “no reasonable alternative” criterion [3]
- Performance enhancement beyond health restoration: The substantial weight loss and potential metabolic effects of GLP-1 RAs could provide competitive advantages beyond restoration of normal health, particularly in weight-category sports [2]
- Diagnostic criteria: Obesity diagnosis is based on BMI ≥ 30 kg/m^2^ or BMI ≥ 27 kg/m^2^ with comorbidities, but many athletes have high BMIs due to muscle mass rather than adiposity, complicating diagnostic assessment [1]

Currently, no systematic data exist on TUE applications or approvals for incretin therapies in competitive athletes [7]. As use of these agents expands, TUE policies and evaluation criteria will require careful consideration to balance legitimate therapeutic needs with anti-doping principles [7].

## 7. Ethical and Regulatory Considerations

### 7.1. Medicalization of Athletic Performance

The use of pharmaceutical agents to enhance athletic performance raises fundamental ethical questions about the nature of sport, fair competition, and the boundaries between therapy and enhancement [1]. Incretin therapies exemplify the blurred distinction between medical treatment and performance enhancement, as they are approved for legitimate medical conditions (T2DM, obesity) but possess properties that could theoretically benefit athletic performance [7].

The medicalization of athletic performance refers to the increasing reliance on medical interventions to optimize training, recovery, and competitive outcomes [1]. While some interventions (e.g., sports medicine, physical therapy, nutritional supplementation) are widely accepted, others (e.g., anabolic steroids, erythropoietin) are prohibited as violations of fair play [1]. The ethical distinction often hinges on whether interventions restore normal function versus enhance beyond natural capacity, although this boundary is inherently ambiguous [1].

For incretin therapies, the medicalization concern centers on use by non-diabetic athletes for weight management and potential metabolic advantages [9]. If GLP-1 RAs become widely used for body composition manipulation in weight-category or aesthetic sports, this could normalize pharmaceutical approaches to weight cutting and undermine emphasis on natural training, nutrition, and discipline [1]. Conversely, if incretin therapies enable safer and more sustainable weight management compared to traditional practices (severe caloric restriction, dehydration, diuretics), they could be viewed as harm-reduction interventions [1].

### 7.2. Accessibility, Equity, and Socioeconomic Disparities

A critical ethical consideration is the accessibility and cost of incretin therapies, which could exacerbate existing socioeconomic disparities in sports [1]. GLP-1 RAs are expensive, with monthly costs ranging from $1000 to $1500 in the United States, although prices vary internationally and insurance coverage may reduce out-of-pocket expenses [2]. For athletes from lower-income backgrounds or countries with limited healthcare resources, access to these agents would be severely constrained [1].

If incretin therapies provide competitive advantages in certain sports, their high cost could create inequities where wealthy athletes or well-funded programs gain advantages unavailable to competitors with fewer resources [1]. This scenario parallels concerns about other expensive performance-enhancing technologies (altitude training facilities, advanced equipment, specialized coaching) that are accessible primarily to elite athletes and well-resourced programs [1].

WADA’s anti-doping framework aims to promote fair competition and protect athlete health, but it does not explicitly address socioeconomic equity in access to permitted substances or technologies [7]. The potential for incretin therapies to exacerbate existing disparities warrants consideration in regulatory decision-making, although practical mechanisms to ensure equitable access are challenging to implement [1].

### 7.3. Athlete Health and Safety

Athlete health and safety are paramount considerations in anti-doping policy, with WADA’s second criterion for substance prohibition explicitly addressing health risks [7]. For incretin therapies, the health implications of use in non-diabetic athletes are poorly characterized due to lack of systematic research in this population [9].

Potential health concerns include:

- Excessive weight loss: Use of GLP-1 RAs by athletes with normal or low body weight could result in unhealthy weight loss, malnutrition, and metabolic complications [9]
- Lean mass loss and sarcopenia: The 20–30% lean mass loss accompanying weight reduction could impair muscle function, increase injury risk, and contribute to long-term sarcopenia [2]
- Gastrointestinal effects: Nausea, vomiting, and diarrhea could impair nutritional intake, hydration status, and training capacity [2]
- Psychological effects: Appetite suppression and altered eating behaviors could contribute to disordered eating patterns, particularly in sports with weight pressures [25]
- Unknown long-term effects: The safety of long-term GLP-1 RA use in healthy, non-diabetic athletes is unknown, as clinical trials have primarily enrolled individuals with obesity or T2DM [2]

Pharmacovigilance data have documented cases of semaglutide misuse for cosmetic weight loss, with adverse events including severe gastrointestinal symptoms, gallbladder disease, and pancreatitis [9]. These reports highlight the potential for harm when GLP-1 RAs are used outside approved indications and without medical supervision [9].

Balancing potential health risks against potential benefits (e.g., safer weight management compared to traditional weight-cutting practices) requires careful consideration of context, individual characteristics, and medical supervision [1]. Prohibition of incretin therapies could drive underground use without medical oversight, potentially increasing health risks [1]. Conversely, permitting use could normalize pharmaceutical weight management and encourage use by athletes who do not meet clinical criteria for treatment [9].

### 7.4. Detection and Enforcement Challenges

Practical considerations regarding detection and enforcement are relevant to anti-doping policy, although they are not formal WADA criteria for substance prohibition [8]. GLP-1 RAs are peptide-based therapeutics that could theoretically be detected in blood or urine samples using immunoassays or mass spectrometry-based methods [26]. However, several challenges complicate detection and enforcement:

- Endogenous GLP-1: Native GLP-1 is an endogenous hormone, requiring differentiation between physiological levels and exogenous administration of GLP-1 RAs [26]
- Structural similarity: Some GLP-1 RAs (e.g., liraglutide) have high structural homology to native GLP-1, complicating analytical differentiation [2]
- Short detection windows: Short-acting GLP-1 RAs (e.g., exenatide twice daily) may have limited detection windows, although long-acting agents (e.g., semaglutide) with week-long half-lives would be more readily detectable [2]
- Lack of reference materials: Development of validated detection methods requires reference standards, proficiency testing, and establishment of decision limits, which have not been developed for incretin therapies [26]

DPP-4 inhibitors, being small-molecule drugs rather than peptides, would be more straightforward to detect using standard analytical methods [12]. However, their weight-neutral effects and lack of clear performance-enhancing properties make them lower priorities for anti-doping surveillance [3].

If incretin therapies were added to the WADA Prohibited List, development and validation of detection methods would be necessary to enable enforcement [8]. The feasibility and cost-effectiveness of such testing would need to be evaluated in the context of prevalence of use and magnitude of performance-enhancing effects [8].

## 8. Discussion

This narrative review has comprehensively evaluated the available evidence regarding incretin-based therapies and their potential classification as doping agents in sports. The analysis reveals a complex landscape where theoretical performance-enhancing mechanisms exist, but clinical evidence of meaningful performance benefits in athletes is lacking [5].

### 8.1. Summary of Key Findings

Molecular and Metabolic Effects: GLP-1 RAs exert pleiotropic effects through GLP-1R activation, including enhanced insulin secretion, appetite suppression, weight loss, cardiovascular protection, and anti-inflammatory actions [3]. Preclinical studies demonstrate that GLP-1R signaling enhances exercise endurance, promotes oxidative muscle fiber transformation, augments mitochondrial biogenesis, and improves glucose uptake through AMPK-dependent pathways [6]. These molecular mechanisms provide a theoretical basis for performance enhancement, particularly in endurance sports.

Body Composition Effects: GLP-1 RAs induce substantial weight loss (15–22.5% with current agents) predominantly from fat mass, with preferential reduction in visceral adipose tissue [2]. However, 20–30% of weight loss comprises lean mass, raising concerns about muscle function and performance capacity [2]. In weight-category sports, this weight loss could enable competition in lower weight classes, but lean mass loss may offset competitive advantages [2].

Clinical evidence in humans: clinical trials have not demonstrated consistent improvements in exercise capacity, VO2 max, or functional performance with GLP-1 RA therapy in humans [5]. The LIPER2 trial found no significant effect of liraglutide on physical performance measures in patients with T2DM [5]. Importantly, existing studies have been conducted in individuals with metabolic disease rather than trained athletes, limiting generalizability [5].

WADA Criteria Assessment: Evaluation against WADA’s three criteria for substance prohibition reveals that incretin therapies partially meet criteria for performance enhancement potential and health risk, but evidence is insufficient to definitively support prohibition [7]. The performance-enhancing potential appears context-dependent and limited, health risks in athletic populations are poorly characterized, and the spirit of sport criterion is ambiguous [7].

Current Regulatory Status: Incretin therapies are not currently listed on the WADA Prohibited List, reflecting the assessment that available evidence does not support prohibition [7]. However, emerging patterns of misuse for cosmetic weight loss and body composition manipulation warrant continued surveillance [9].

### 8.2. Gaps in Current Evidence

Several critical knowledge gaps limit definitive conclusions about incretin therapy as doping:

1. Lack of studies in athletes: No published studies have evaluated the effects of GLP-1 RAs on athletic performance, training adaptations, or sport-specific outcomes in competitive athletes or highly trained individuals [5].
2. Translation from preclinical to clinical: The endurance-enhancing effects observed in rodent models have not been replicated in human studies, raising questions about species differences, dosing, and relevance to athletic performance [6].
3. Long-term effects: The safety and efficacy of long-term GLP-1 RA use in healthy, non-diabetic athletes are unknown, as clinical trials have primarily enrolled individuals with obesity or T2DM for durations of 1–2 years [2].
4. Interaction with training: The effects of GLP-1 RAs on training adaptations, recovery, and performance when combined with structured exercise programs are poorly understood [10].
5. Prevalence of use: No systematic data exist on the prevalence, patterns, or motivations for incretin therapy use among athletes, limiting assessment of the scope of potential misuse [9].
6. Detection methods: Validated analytical methods for detecting GLP-1 RAs in anti-doping samples have not been developed or implemented [26].

### 8.3. Comparison with Other Substance Classes

Comparing incretin therapies with other substance classes on the WADA Prohibited List provides context for regulatory decision-making:

Anabolic Steroids (S1): Anabolic-androgenic steroids demonstrate clear, dose-dependent increases in muscle mass, strength, and power, with extensive evidence of misuse in sports and significant health risks [1]. Unlike incretin therapies, anabolic steroids have no legitimate therapeutic use in most athletes and are universally prohibited [1].

Erythropoietin (S2): Recombinant erythropoietin (EPO) increases red blood cell mass and oxygen-carrying capacity, providing clear performance benefits in endurance sports [1]. EPO misuse is well-documented in cycling and other endurance sports, and detection methods are established [1]. In contrast, incretin therapies lack comparable evidence of performance enhancement or prevalence of misuse [5].

Beta-2 Agonists (S3): Inhaled beta-2 agonists are permitted for athletes with asthma under TUE provisions, while systemic use is prohibited due to potential anabolic and performance-enhancing effects [7]. This regulatory approach balances therapeutic needs with anti-doping principles, providing a potential model for incretin therapies if evidence of misuse emerges [7].

Diuretics (S5): Diuretics are prohibited primarily as masking agents and for rapid weight loss in weight-category sports [7]. GLP-1 RAs could theoretically serve similar functions (weight manipulation), but their gradual onset and lean mass loss distinguish them from acute diuretic effects [2].

### 8.4. Future Research Directions

To inform evidence-based anti-doping policy regarding incretin therapies, several research priorities are recommended:

7. Performance studies in athletes: Randomized controlled trials evaluating the effects of GLP-1 RAs on sport-specific performance outcomes, training adaptations, and body composition in competitive athletes across various disciplines [5].
8. Mechanistic studies: Investigation of molecular mechanisms underlying potential performance effects, including muscle fiber-type composition, mitochondrial function, substrate utilization, and recovery markers [6].
9. Safety and tolerability: Assessment of adverse effects, nutritional status, and long-term health outcomes in non-diabetic athletes using GLP-1 RAs for weight management or performance enhancement [9].
10. Prevalence and patterns of use: Epidemiological studies characterizing the prevalence, motivations, and patterns of incretin therapy use among athletes in different sports and competitive levels [9].
11. Detection method development: Development and validation of analytical methods for detecting GLP-1 RAs and DPP-4 inhibitors in anti-doping samples, including establishment of decision limits and reference populations [26].
12. Combination with training interventions: Studies evaluating the interaction between GLP-1 RAs and structured exercise programs (resistance training, endurance training) on body composition, performance, and health outcomes [10].

### 8.5. Limitations of This Review

This narrative review has several limitations that should be acknowledged. First, the literature search was limited to specific databases and may not have captured all relevant publications, particularly gray literature, conference abstracts, or non-English language publications [2]. Second, the heterogeneity of study designs, populations, and outcome measures precluded quantitative meta-analysis, limiting the strength of conclusions [2]. Third, the rapid evolution of incretin-based therapies, with new agents and indications emerging frequently, means that evidence may become outdated quickly [2]. Fourth, the lack of studies specifically in athletic populations necessitated extrapolation from clinical trials in individuals with obesity or T2DM, which may not reflect effects in healthy, trained athletes [5].

## 9. Conclusions

Based on a comprehensive evaluation of available evidence, incretin-based therapies including GLP-1 receptor agonists and DPP-4 inhibitors do not currently meet the criteria for classification as doping agents under WADA regulations. While these agents possess theoretical performance-enhancing properties through effects on body composition, energy metabolism, and cardiovascular function, clinical evidence of meaningful performance benefits in athletes is lacking.

The substantial weight loss induced by GLP-1 RAs could provide competitive advantages in weight-category sports, but concurrent lean mass loss (20–30% of total weight loss) may offset these benefits and could be detrimental in strength and power sports. Preclinical studies demonstrating endurance-enhancing effects have not been replicated in human clinical trials, and no studies have been conducted in competitive athletes.

Current evidence suggests that incretin therapies partially meet WADA criteria for performance enhancement potential and health risk, but the magnitude of effects, context-dependency, and lack of data in athletic populations preclude definitive prohibition. The spirit of the sport criterion is ambiguous, with reasonable arguments both for and against classification as violations of sporting ethics.

Several considerations support continued surveillance and potential future re-evaluation:

13. Emerging misuse patterns: Pharmacovigilance data document increasing off-label use of GLP-1 RAs for cosmetic weight loss, suggesting potential for misuse in athletic contexts.
14. Theoretical performance-enhancing mechanisms: Molecular and preclinical evidence of endurance enhancement, metabolic optimization, and body composition effects warrant monitoring as use patterns evolve.
15. Accessibility and equity concerns: The high cost of GLP-1 RAs could exacerbate socioeconomic disparities if these agents provide competitive advantages.
16. Evolving therapeutic landscape: New dual and triple agonists (e.g., tirzepatide) with enhanced efficacy may have different performance implications than current GLP-1 RAs.

Recommendations for stakeholders include:

For WADA and anti-doping organizations: Continue monitoring prevalence and patterns of incretin therapy use among athletes; commission research on performance effects in athletic populations; and develop detection methods to enable enforcement if future evidence supports prohibition.

For sports medicine practitioners: Provide evidence-based guidance to athletes regarding appropriate use of incretin therapies for legitimate medical indications; discourage off-label use for performance enhancement or weight management in the absence of clinical indications; and monitor for adverse effects and nutritional adequacy in athletes using these agents.

For athletes and coaches: Recognize that current evidence does not support use of incretin therapies for performance enhancement; prioritize evidence-based training, nutrition, and recovery strategies; and consult with medical professionals before using any pharmaceutical agents.

For researchers: Conduct rigorous studies evaluating the effects of incretin therapies on athletic performance, training adaptations, body composition, and health outcomes in competitive athletes; investigate mechanisms underlying potential performance effects; and assess prevalence and motivations for use in athletic populations.

In conclusion, while incretin-based therapies possess theoretical properties that could influence athletic performance, current evidence does not support their classification as doping agents. However, the rapidly evolving landscape of incretin pharmacology, emerging patterns of off-label use, and gaps in knowledge regarding effects in athletes warrant continued vigilance and research to inform evidence-based anti-doping policy.

## Data Availability

No new data were created or analyzed in this study. Data sharing is not applicable to this article.
